# Novel Montmorillonite/TiO_2_/MnAl-Mixed Oxide Composites Prepared from Inverse Microemulsions as Combustion Catalysts

**DOI:** 10.3390/ma10111326

**Published:** 2017-11-19

**Authors:** Bogna D. Napruszewska, Alicja Michalik-Zym, Melania Rogowska, Elżbieta Bielańska, Wojciech Rojek, Adam Gaweł, Monika Wójcik-Bania, Krzysztof Bahranowski, Ewa M. Serwicka

**Affiliations:** 1 Jerzy Haber Institute of Catalysis and Surface Chemistry, Niezapominajek 8, 30-239 Krakow, Poland; ncnaprus@cyf-kr.edu.pl (B.D.N.); ncmichal@cyf-kr.edu.pl (A.M.-Z.); melania.rogowska@gmail.com (M.R.); ncbielan@cyf-kr.edu.pl (E.B.); ncrojek@cyf-kr.edu.pl (W.R.); 2 Faculty of Geology, Geophysics and Environmental Protection, AGH University of Science and Technology, al. Mickiewicza 30, 30-059 Krakow, Poland; agawel@agh.edu.pl (A.G.); wojcikm@agh.edu.pl (M.W.-B.); bahr@agh.edu.pl (K.B.)

**Keywords:** montmorillonite/hydrotalcite composite, montmorillonite/titania composite, organoclay, inverse micelle, Mn-Al mixed oxide, combustion catalysts

## Abstract

A novel design of combustion catalysts is proposed, in which clay/TiO_2_/MnAl-mixed oxide composites are formed by intermixing exfoliated organo-montmorillonite with oxide precursors (hydrotalcite-like in the case of Mn-Al oxide) obtained by an inverse microemulsion method. In order to assess the catalysts’ thermal stability, two calcination temperatures were employed: 450 and 600 °C. The composites were characterized with XRF (X-ray fluorescence), XRD (X-ray diffraction), HR SEM (high resolution scanning electron microscopy, N_2_ adsorption/desorption at −196 °C, and H_2_ TPR (temperature programmed reduction). Profound differences in structural, textural and redox properties of the materials were observed, depending on the presence of the TiO_2_ component, the type of neutralization agent used in the titania nanoparticles preparation (NaOH or NH_3_ (aq)), and the temperature of calcination. Catalytic tests of toluene combustion revealed that the clay/TiO_2_/MnAl-mixed oxide composites prepared with the use of ammonia showed excellent activity, the composites obtained from MnAl hydrotalcite nanoparticles trapped between the organoclay layers were less active, but displayed spectacular thermal stability, while the clay/TiO_2_/MnAl-mixed oxide materials obtained with the aid of NaOH were least active. The observed patterns of catalytic activity bear a direct relation to the materials’ composition and their structural, textural, and redox properties.

## 1. Introduction

Widespread emission of volatile organic compounds (VOCs) to the atmosphere, occurring chiefly as a result of industrial and transportation activities, is regarded as a major global environmental hazard, due to the toxic, mutagenic, and carcinogenic nature of the pollutants, and to their role in the formation of the photochemical smog. Among the different techniques for abatement of VOCs, neutralization based on catalytic combustion is considered particularly appropriate, owing to its low operational costs and high destruction efficiency [1]. Catalyst design is usually based on noble metals or on transition metal oxides, the latter being attractive due to their lower price. It has been repeatedly demonstrated that efficient VOCs combustion catalysts can be developed from clay minerals of anionic and/or cationic character (e.g., [2,3,4,5,6,7,8,9,10,11,12,13,14,15,16,17,18,19]). Recently, we proposed a novel strategy for preparing VOCs combustion catalysts, in which the catalytically active Mn-Al oxide nanoparticles, obtained from hydrotalcite (Ht) precursor synthesized by an inverse microemulsion method, are trapped between randomly oriented layers of smectite (Laponite) [19]. In such a composite the active phase forms uniform grains of nanometer dimensions, well separated by clay layers, which hinders sintering phenomena. Moreover, termination of clay basal faces with the chemically inert silica sheet prevents catalyst deactivation via reaction with the support. 

In the present work we propose further modification of the catalyst design by incorporation of Mn-Al inverse micelles into the smectite matrix loaded previously with titania. TiO_2_ belongs to the most frequently studied catalyst supports, and in many cases it has been shown that its use is beneficial for catalytic activity in total oxidation reactions [20,21,22,23,24]. However, rather than using conventional microporous Ti-pillared clay (Ti-PILC), we employed novel materials, recently developed in our laboratory, referred to as Ti-IMEC composites, formed from exfoliated organo-montmorillonite and inverse microemulsion containing Ti oxo-hydroxy species [25]. The composites contain larger TiO_2_ particles and are therefore mesoporous, which makes them texturally more suitable for entrapment of inverse micelles with Mn-Al Ht-like precursor. In the present study montmorillonite was chosen as the clay component. Toluene was used as a model VOC compound.

## 2. Materials and Methods 

### 2.1. Materials

Active phase precursor in the form of MnAl Ht-like nanoparticles was prepared following the double-microemulsion method proposed by Bellezza et al. [26] and described in detail previously [19]. Inverse microemulsion consists of very small droplets of water dispersed in a continuous oil phase. The water droplets are stabilized by the presence of surfactant and co-surfactant. When two microemulsions, each containing reactants required for Ht formation, are mixed, the intermicellar exchange of solutes leads to product precipitation within the limited space of the aqueous micellar core, yielding nano-size Ht particles. Briefly, two inverse microemulsions of Ht-forming reagents were prepared, one from the aqueous solution of Mn(NO_3_)_2_ and Al(NO_3_)_3_, with the intended Mn:Al = 3, the other from NH_3_ (aq). Each aqueous solution was dispersed in the organic medium based on isooctane as oil phase, cetyltrimethylammonium bromide (CTABr) as surfactant, and n-butanol as co-surfactant, and stirred till the mixtures turned into stable transparent liquids. Equal volumes of the two microemulsions were mixed at 70 °C to enable precipitation of the Ht-like phase, referred to as MnAl(im). The suspensions of MnAl(im) precursor in the organic mother liquor were further used for the preparation of composites. For the sake of comparison, the Ht-like material of similar stoichiometry was prepared by the standard co-precipitation method and is denoted MnAl(st).

The starting montmorillonite clay used for the preparation of composites was the sodium form of the less than 2 μm particle size fraction separated by sedimentation from Jelšový Potok bentonite (Slovakia), denoted Na-mt. The material was transformed into an organic derivative, referred to as CTA-Mt, by a routine cation exchange procedure with the CTABr aqueous solution. 

The TiO_2_-containing catalysts were obtained via Ti-IMEC intermediates prepared following the procedure described in detail previously [25]. Briefly, the CTA-Mt form was subjected to an exfoliation treatment [27] by dispersing in 1-hexanol and ultrasonication. Inverse Ti-containing microemulsion was prepared from an aqueous solution of Ti species obtained by controlled hydrolysis of TiCl_4_ [28] added to 1-hexanol as oil phase and CTABr as surfactant. The system was stirred till the mixture turned into a stable transparent liquid. Thus formed Ti-containing microemulsion was added drop-wise to CTA-Mt dispersion in 1-hexanol, at 60 °C, in the amount providing a Ti loading of ca. 40 wt%. Subsequently, the pH was raised to 7 either by drop-wise addition of 1 M NaOH as described in reference [25] and the dispersion stirred for further 0.5 h. Alternatively, to avoid introduction of sodium into the composite material, raising of the pH was carried out using 25% NH_3_ (aq) solution. The product obtained by neutralization with NaOH is further referred to as Ti-IMEC_NaOH_, the one neutralized with ammonia, as Ti-IMEC_NH3_. The final composites were obtained by mixing Ti-IMEC_NaOH_ or Ti-IMEC_NH3_ with MnAl(im), all components used as suspensions in their organic mother liquors. For each Ti-IMEC preparative route (NaOH or NH_3_) composites with two different MnAl(im) contents were obtained, the intended loadings corresponding approximately to 1:9 and 1:3 MnAl(im)/Ti-IMEC weight ratios. The mixtures were vigorously stirred for 18 h, followed by 1 h ultrasonication. Finally, the precipitates were washed, initially with a 1:1 mixture of chloroform and ethanol, followed by 1:1 mixture of ethanol and water, ending with distilled water. The products were dried by lyophilization and calcined for 4 h at 450 °C or 600 °C. The catalysts were denoted MnAl(im)/Ti-IMEC_NaOH_-x-temp, or MnAl(im)/Ti-IMEC_NH3_-x-temp, where “x” was I or II, depending on the load of MnAl(im) component, and “temp” was the temperature of calcination, i.e., the sample signature MnAl(im)/Ti-IMEC_NaOH_-II-450 means that the catalyst was obtained from Ti-IMEC neutralized with NaOH, containing larger loading of MnAl(im) component and was calcined at 450 °C. 

In order to check on the role of titania component, a composite without TiO_2_ addition was obtained, according to the procedure described in reference [19], by combining suspensions of organoclay with MnAl(im). Briefly, the CTA-Mt was dispersed in isopropanol and added to the as received suspension of MnAl(im) in organic mother liquor, in relative amounts corresponding to the higher of the two active phase loadings used in the preparation of TiO_2_-containing composites. After vigorous stirring at 20 °C the composite was filtered off, lyophilized and calcined for 4 h at 450 °C or 600 °C. The resulting catalysts are referred to as MnAl(im)/CTA-Mt-450 and MnAl(im)/CTA-Mt-600. Finally, for the sake of assessing the purposefulness of the catalyst synthesis from inverse microemulsion/organoclay mixture, yet another composite was prepared, of the clay/active phase proportion similar to that of MnAl(im)/CTA-Mt, this time via a simple totally inorganic route, by mixing appropriate amounts of aqueous suspension of MnAl hydrotalcite, obtained by the standard co-precipitation method, and referred to as MnAl(st), and the aqueous dispersion of the sodium form of montmorillonite, Na-Mt. Both components were stirred vigorously, filtered off, lyophilized and calcined at 450 and 600 °C for 4 h. The samples are referred to as MnAl(st)/Na-Mt-450 and MnAl(st)/Na-Mt-600.

### 2.2. Methods

Powder X-ray diffraction (XRD) patterns were recorded using X’Pert PRO MPD diffractometer (PANalytical, Holland) with CuKα radiation. The crystallite sizes of anatase modification of TiO_2_ were estimated as an average of Scherrer calculations carried out for the (101) and (200) peaks, and of Mn_3_O_4_ for the (112), (103), and (211) reflections.

Chemical composition of the investigated solids was determined with a ZSX Primus II spectrometer (Rigaku, Japan) with a Rh anode as X-ray source, using a calibration based on certified reference materials. 

High magnification SEM images were recorded for the uncoated samples deposited on 200 Mesh copper grids covered with carbon support film, using a JSM-7500F Field Emission Scanning Electron Microscope (SEM) (JEOL, Japan). 

Temperature programmed reduction (TPR) was performed in a quartz U-shaped tubular reactor (home-made). About 0.015 g of sample was used. The reducing gas was a mixture of 5 vol% H_2_ in Ar (Linde, H_2_ 5% in Ar), at a total flow rate of 30 mL·min^−1^. The temperature was increased at a rate of 10 °C·min^−1^ from room temperature to 700 °C. The TPR profiles were recorded using a thermal conductivity detector (TCD).

Textural parameters were derived from N_2_ adsorption/desorption measurements performed at −196 °C with the use of an AUTOSORB 1 instrument (Quantachrome Instruments, USA). Prior to measurement, the samples were outgassed at 200 °C for 3 h. Specific surface areas were calculated according to the Brunauer–Emmett–Teller method (S_BET_) in the relative pressure range 0.02–0.04. The total pore volume (V_tot_) was calculated from the amount of N_2_ adsorbed at a relative vapor pressure p/p_0_ = 0.996. The mean diameters of all pores (D^av^) were evaluated using the Gurvitch formula D^av^ = 4V_tot_/S_BET_. Pore size distribution (PSD) profiles were determined by the NL DFT method.

Catalytic combustion of toluene was carried out in a fixed-bed flow quartz reactor of 10 mm inner diameter, loaded with ca. 0.5 g of a catalyst (particle size 0.3–0.5 mm), in the temperature range 100–400 °C. Toluene at 500 ppm concentration was fed to the flow by passing air through a saturator, at GHSV of 10,000 h^−1^. The only reaction products were CO_2_ and water. Toluene consumption was measured by GC-FID (SRI 8610A) and CO_2_ evolution by GC-FID (SRI 310). 

## 3. Results and Discussion

### 3.1. Characterization of Composites

The chemical composition of the synthesized materials is shown in Table 1. The data indicate that in the titania-containing composites the clay component constitutes about half of the total catalyst weight, with a Ti/Si atomic ratio of ca. 1. Moreover, the analysis reveals that the catalysts based on Ti-IMEC_NaOH_, obtained with the use of NaOH during the neutralization step, contain a significant amount of sodium. This information was the incentive for the preparation of the titania-clay component by a modified procedure, using ammonia solution as the neutralizing agent. The resulting Ti-IMEC_NH3_ derived composites do not contain Na, and maintain a Mn content comparable to the Ti-IMEC_NaOH_ based counterparts. The titania-free composites have a similar content of Mn to the Ti-IMEC-based composites. The MnAl(st)/Na-Mt sample contains some sodium stemming from the clay component, while the MnAl(im)/CTA-Mt, obtained from the organoclay, is Na-free.

**Figure 1:**
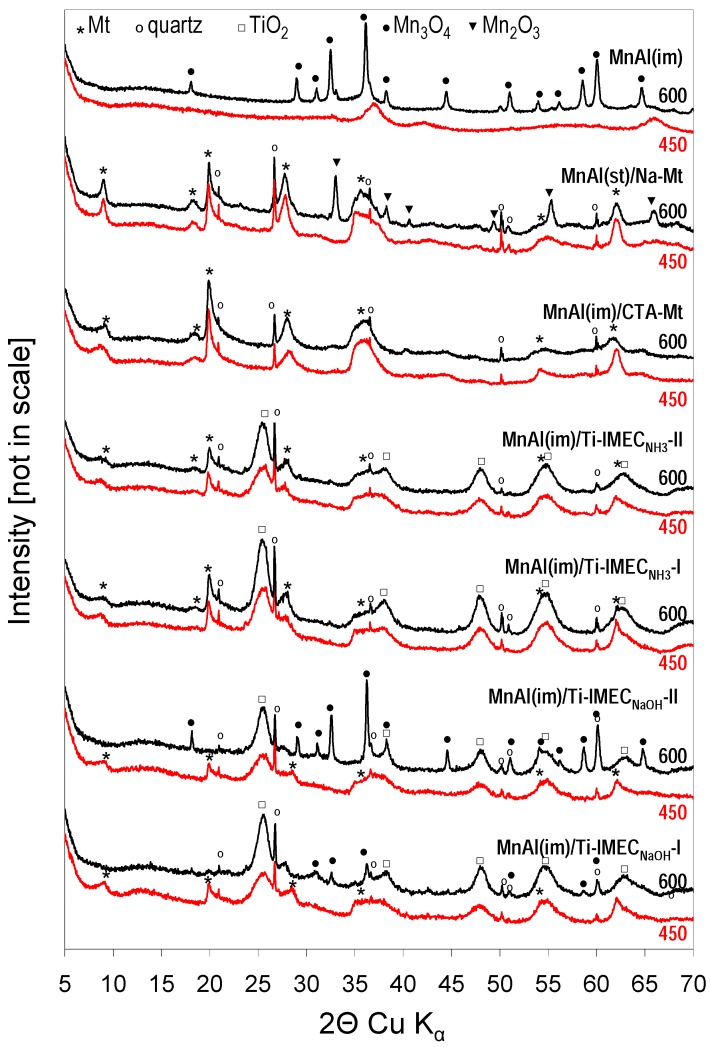
Powder X-ray diffraction (XRD) patterns of investigated composites and the MnAl(im) active phase calcined at 450 and 600 °C.

XRD diagrams of composite catalysts calcined at 450 and 600 °C are collated in Figure 1. In addition, the pattern of MnAl(im) calcined at 450 and 600 °C is shown, to facilitate identification of reflections associated with the Mn-Al mixed oxide active phase. After calcination at 450 °C, the Mn-Al mixed oxide phase shows several very broad features, pointing to the formation of highly amorphized ε-MnO_2_ (ref. code 30-0820) known to be catalyzed by the presence of aluminum [29], while after treatment at 650 °C, a clear set of peaks characteristic of well crystallined Mn_3_O_4_ (ref. code 24-0734) emerges. The XRD patterns of TiO_2_-free MnAl(im)/CTA-Mt-450 and MnAl(im)/CTA-Mt-600 materials are relatively simple, as the only well resolved features can be identified as belonging to calcined montmorillonite and the quartz impurity. The position of the low intensity (001) reflection of montmorillonite corresponds to the interplanar distance d_001_ ≈ 1.0 nm, the value characteristic of clay with thermally collapsed layers. In fully exfoliated clay with the house of cards structure this reflection should be absent, therefore its appearance indicates that partial restacking of exfoliated clay layers occurred. No reflection that might be attributed to a Mn-bearing oxide phase is visible, even after calcination at 600 °C. This result is at variance with the behavior of analogical composites based on Laponite, where upon calcination at 600 °C crystallization of Mn_3_O_4_ was observed [19]. The result suggests that the layers of synthetic Laponite offer a more efficient thermal barrier than those of montmorillonite, so that the heat evolved upon combustion of interlayer CTA cations in Laponite matrix is more likely to generate hotspots facilitating crystallization of Mn_3_O_4_. It has been proposed that one of the reasons for differences in thermal properties of organoclays based on Laponite, as opposed to montmorillonite, is that in the synthetic Laponite the silica sheets adhering to the organic matter are free of heteroatom impurities, while in montmorillonite the tetrahedral sheet properties are modified by the Al for Si substitution [30]. The MnAl(st)/Na-Mt-450 sample obtained by the inorganic route also displays only the features of thermally collapsed montmorillonite and quartz impurity, but in the MnAl(st)/Na-Mt-600 material additional reflections appear, pointing to the crystallization of the Mn_2_O_3_ phase. 

In all Ti-IMEC based samples the formation of the anatase modification of TiO_2_ (ref. code 21-1272) is observed already after calcination at 450 °C (Figure 1). Titania crystallinity is improved upon calcination at 600 °C, where, according to Scherrer calculations, the coherently scattering domains of anatase reach ca. 6–7 nm. Analysis of the XRD patterns of Ti-IMEC based composites shows that in all samples calcined at 450 °C, whether prepared with NH_3_ or NaOH, only features attributable to montmorillonite, anatase, and quartz impurity are visible, with no evidence of any Mn-containing oxide phase. Also, no crystalline Na-containing phase could be detected in samples obtained from Ti-IMEC_NaOH_. This shows that sodium either enters the clay interlayer, and/or becomes incorporated into anatase, and/or forms amorphous sodium titanate-like phase. The response of Ti-IMEC_NH3_ and Ti-IMEC_NaOH_ derived composites to thermal treatment at 600 °C is different. In the former the evidence of Mn-containing oxide crystallization is hard to find, while in MnAl(im)/Ti-IMEC_NaOH_-I-600 and, especially, in MnAl(im)/Ti-IMEC_NaOH_-II-600, sharp reflections of Mn_3_O_4_ appear. Moreover, in MnAl(im)/Ti-IMEC_NaOH_-I-600 and in MnAl(im)/Ti-IMEC_NaOH_-II-600, the features related to montmorillonite disappear. This points to the loss of the long range order in the clay component and shows that the Ti-IMEC_NaOH_ based composites possess lower structural stability than the Ti-IMEC_NH3_ derived ones. Both effects, i.e., the more facile degradation of clay and the ease of Mn_3_O_4_ crystallizatiom are likely to be due to the presence of sodium, whose compounds are known to act as fluxes enhancing solid state transformations [31,32]. It should be noted, however, that according to previous study [25], in the absence of MnAl(im) component no destruction of clay layers occurs in Ti-IMEC_NaOH_ calcined at 600 °C, showing that the former must also be involved in the observed structural transformation. The estimated average crystal size of Mn_3_O_4_ in MnAl(im)/Ti-IMEC_NaOH_-II-600 is ca. 55–60 nm. 

**Figure 2:**
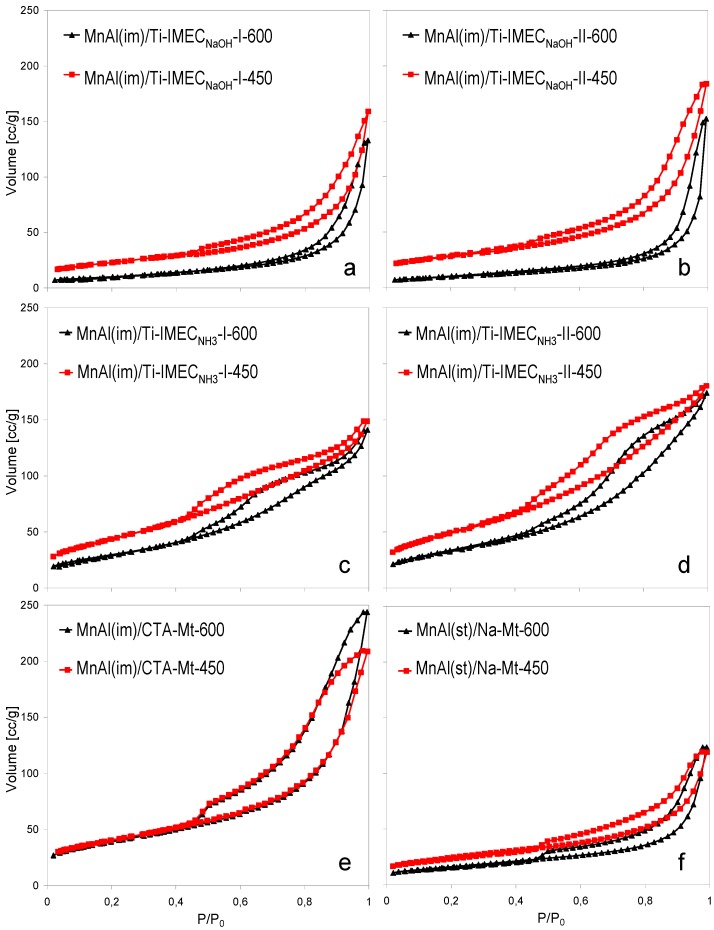
N_2_ adsorption/desorption isotherms of investigated composites calcined at 450 and 600 °C, (**a**) MnAl(im)/Ti-IMEC_NaOH_-I; (**b**) MnAl(im)/Ti-IMEC_NaOH_-II; (**c**) MnAl(im)/Ti-IMEC_NH3_-I; (**d**) MnAl(im)/Ti-IMEC_NH3_-II; (**e**) MnAl(im)/CTA-Mt; (**f**) MnAl(st)/Na-Mt.

N_2_ adsorption/desorption isotherms obtained for the composite catalysts calcined at 450 and 600 °C are presented in Figure 2, and the textural parameters are gathered in Table 2. 

Analysis of isotherms was carried out based on the recent IUPAC report [33]. All isotherms may be classified as Type II, and most of the accompanying hysteresis loops are of type H3 (Figure 2a,b,e,f). The strong upward swing of these isotherms is the result of unrestricted monolayer-multilayer adsorption up to high p/p_0_. Such an isotherm shape indicates that the material contains both mesopores, which are responsible for the hysteresis, and macropores, which adsorb nitrogen in the relative pressure range of 0.98–1.00. In the case of catalysts derived from Ti-IMEC_NH3_, the hysteresis loops are of a more complex shape. In MnAl(im)/Ti-IMEC_NH3_-II-450 and MnAl(im)/Ti-IMEC_NH3_-II-600 (Figure 2d) desorption branches display a two-step profile, encountered in materials containing a contribution from mesopores with partially plugged passages [34,35]. The desorption step at higher relative pressure reflects emptying of open mesopores, while the blocked pores remain filled until the relative pressure drops below p/p_0_ ~0.5 and the cavitation of the nitrogen condensate leads to N_2_ desorption from the confined areas. Such hysteresis loops are referred to as type H5. Samples MnAl(im)/Ti-IMEC_NH3_-I-450 and MnAl(im)/Ti-IMEC_NH3_-I-600 (Figure 2c) show also features of a two-step pore emptying, although less pronounced, and shifted to lower p/p_0_. Therefore, the resulting hysteresis loops are intermediate between H3 and H5 type, indicating that pore blocking effects play a role also in these samples.

The isotherms of TiO_2_-free MnAm(im)/CTA-Mt-450 and MnAm(im)/CTA-Mt-600 samples are of type II with H3 hysteresis loops (Figure 2e), and are very similar to those reported for analogical composites based on Laponite [19]. The exceptionally good thermal stability of the material, evidenced by the virtual lack of impact of the calcination temperature on the isotherm character and position, is another feature common with Laponite-derived composites [19]. The MnAm(st)/Na-Mt-450 and MnAm(st)/Na-Mt-600 composites, obtained by an inorganic route, have much lower adsorption capacity and poorer thermal stability than the MnAm(im)/CTA-Mt-450 and MnAm(im)/CTA-Mt-600 counterparts, as manifested by the position of their isotherms (Figure 2f) and the textural parameters given in Table 2. All titania-containing composites also show a downward shift of the isotherms upon increase of the calcination temperature from 450 to 600 °C. It may be presumed that thermal evolution of the titania component, visible in the XRD patterns, is a factor contributing to the observed changes of the composites’ texture. The effect of textural shrinkage is particularly visible in the case of composites based on Ti-IMEC_NaOH_ (Figure 2, Table 2), whose specific surface area after calcination at 600 °C is reduced by over 50%, to be compared with ca. 30% reduction observed for Ti-IMEC_NH3_ derived samples. The fall of the specific surface is accompanied by an increase of the average pore diameter of composites calcined at 600 °C, which, again, is particularly strong for Ti-IMEC_NaOH_ related composites (Table 2). 

**Figure 3:**
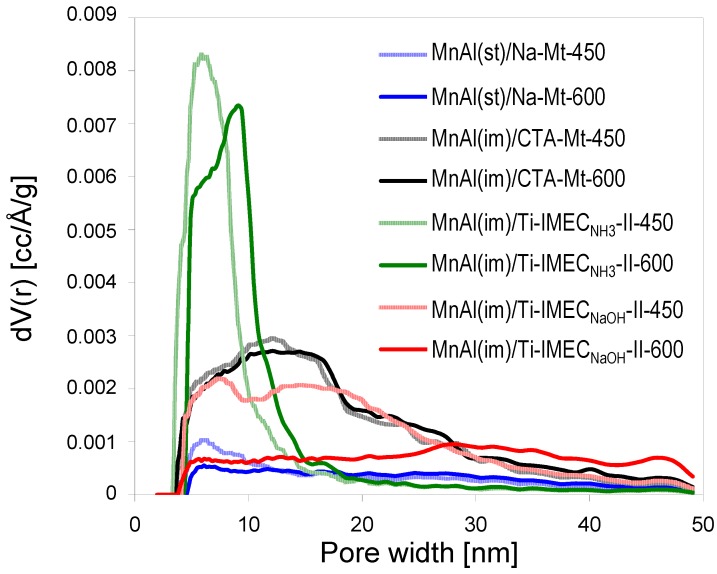
Pore size distribution profiles of investigated composites calcined at 450 and 600 °C.

Evolution of PSD profiles in composites with comparable loading of the active phase upon increase of the calcination temperature also shows evident differences between the materials (Figure 3). Thus, the MnAl(im)/Ti-IMEC_NH3_-II-450 sample has a narrow pore size distribution (PSD), shifted to slightly higher pore widths after calcination at 600 °C. In contrast, the PSD of MnAl(im)/Ti-IMEC_NaOH_-II-450 is much broader and bimodal, while in MnAl(im)/Ti-IMEC_NaOH_-II-600 most of the porosity below pore size 250 nm vanishes. The PSD of the titania-free MnAl(im)/CTA-Mt material is almost independent of the calcination temperature, which confirms that it is the recrystallization of TiO_2_ nanoparticles that contributes to the temperature-induced textural changes in titania-bearing materials. However, the accelerated textural collapse of MnAl(im)/Ti-IMEC_NaOH_ samples indicates that yet another factor influences the thermal evolution of these materials. Since the effect coincides with the XRD data pointing to the destruction of the clay lattice upon thermal treatment, the textural caving-in is attributed to the flux-like action of sodium present in these materials, causing local softening of the solid components and closure of the pore network.

**Figure 4:**
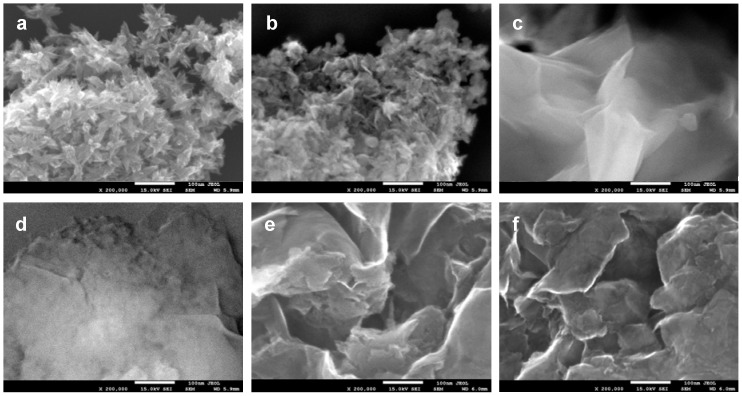
High resolution (HR) SEM images of (**a**) precipitate obtained from neutralized micellar TiO_2_ precursor; (**b**) as received MnAl(im); (**c**) CTA-Mt exfoliated in 1-hexanol; (**d**) MnAl(im)/Ti-IMEC_NaOH_-II-600; (**e**) MnAl(im)/Ti-IMEC_NH3_-II-600; (**f**) MnAl(im)/CTA-Mt-600.

High magnification SEM images of selected uncoated composites and components used for their synthesis are gathered in Figure 4. Thus, Figure 4a,b shows the morphology of the precipitate obtained from neutralized micellar TiO_2_ precursor and of the as received MnAl(im), respectively. In both cases the powders are composed of fine, uniform particles formed under the influence of spatial constraints exerted by the limited volume of inverse micelles. Figure 4c shows the fluffy house of cards texture of exfoliated CTA-Mt deposited on the copper grid from dispersion in 1-hexanol. Figure 4d–f shows, respectively, the images of MnAl(im)/Ti-IMEC_NaOH_-II-600, MnAl(im)/Ti-IMEC_NH3_-II-600, and MnAl(im)/CTA-Mt-600 composite materials. It is visible that the appearance of both latter materials (Figure 4e,f) is determined by the loose, haphazard arrangement of platey clay particles, resembling that of CTA-Mt (Figure 4c). No obvious agglomerates of introduced nanoparticles of TiO_2_ and/or Mn-Al mixed oxide are present. In contrast, in MnAl(im)/Ti-IMEC_NaOH_-II-600 (Figure 4d) areas exist where the clay particles appear to fuse with the agglomerates of nanoparticles of oxide components. The image is consistent with the results of XRD and textural analyses indicating that Ti-IMEC_NaOH_ are thermally less stable and upon calcination at 600 °C both the structure and the texture of the materials collapses. One may envisage that the growing structural disorder is associated with softening of the layers whose partial merger leads to strong modification of the materials texture. The occurrence of nanoparticle agglomerates may also be the reason for the appearance in MnAl(im)/Ti-IMEC_NaOH_-II-600 of Mn_3_O_4_ with crystal size (55–60 nm) exceeding the thickness of a single primary MnAl(im) particle (Figure 4b).

**Figure 5:**
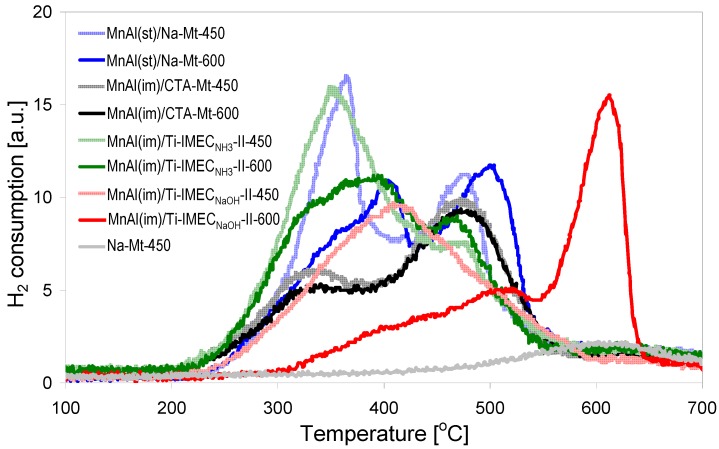
H_2_ temperature programmed reduction (TPR) profiles of investigated composites calcined at 450 and 600 °C.

Reducibility of the catalysts, an important factor influencing the material’s activity in total oxidation processes, was assessed by means of temperature programmed reduction (TPR) with hydrogen. Figure 5 shows the TPR profiles recorded for samples with similar Mn content, calcined at 450 or 600 °C. All major hydrogen consumption effects are due to the reduction of manganese-containing oxide phases. Of the other composite components, in the investigated temperature range only montmorillonite clay shows a very weak maximum around 600 °C, attributed to the reduction of structural iron (Figure 5). Transformation of manganese oxides upon reduction proceeds according to the general scheme: MnO_2_ → Mn_2_O_3_ → Mn_3_O_4_ → MnO [36]. Inspection of Figure 5 shows that TPR curves differ significantly depending on the type of composites. In addition, in most cases the course of reduction depends visibly on the temperature of calcination, because MnOx oxides are known to lose lattice oxygen upon increasing the temperature of thermal treatment in air [37,38]. In most samples the TPR profiles are essentially bimodal. The low temperature maxima occurring below 400 °C are attributed to the reduction of Mn^4+^, present in MnO_2_-like species and/or as defects in oxygen-rich surface layers of other MnOx forms [19,36,37,38,39], to Mn^3+^ in Mn_2_O_3_-like stoichiometry. The subsequent reduction step of Mn_2_O_3_ to Mn_3_O_4_ is usually not distinguishable as a separate maximum, because it overlaps with the former effect [36]. The second maximum, occurring in the 450–520 °C temperature range, is assigned to the consecutive reduction of Mn_3_O_4_ to MnO. The situation in which the intensity of the first maximum prevails over the high temperature effects, as in the case of MnAl(im)/Ti-IMEC_NH3_-II-450, MnAl(im)/Ti-IMEC_NH3_-II-450 composites and the MnAl(st)/Na-Mt-450 sample prepared by the inorganic route, is indicative of a substantial content of MnO_2_ [39]. This conclusion is corroborated by the H/Mn ratio found for these materials (Table 2). The theoretical hydrogen consumption for MnO_2_ corresponds to H/Mn = 2, for Mn_2_O_3_ to H/Mn = 1 and for Mn_3_O_4_ to H/Mn ≈ 0.7. In the case of MnAl(im)/Ti-IMEC_NH3_-II-450, MnAl(im)/Ti-IMEC_NH3_-II-450, and MnAl(st)/Na-Mt-450 the observed H/Mn ratios equal 1.6, 1.4, and 1.5, respectively, which is meaningfully higher than the value expected for Mn_2_O_3_ reduction. 

The H/Mn ratio is also quite high in the MnAl(st)/Na-Mt-600 sample, whose XRD pattern points to crystallization of Mn_2_O_3_. This means, that apart from the sesquioxide detected by XRD analysis, the material also contains Mn^4+^ species, which are responsible for the initial low temperature rise of the TPR profile, while the shift of the first maximum to higher temperature (around 400 °C) reflects the contribution from the reduction of the crystalline Mn_2_O_3_ component. The TPR profiles of the titania-free MnAl(im)/CTA-Mt-450 and MnAl(im)/CTA-Mt-600 samples are very similar, showing that the composite changes very little upon increase of the calcination temperature. The first maximum is lower than the high temperature one, indicating a lower content of Mn^4+^ species than in the MnAl(im)/Ti-IMEC_NH3_-II composite which contains addition of titania, and in the MnAl(st)/Na-Mt sample prepared by the inorganic route. The H/Mn ratio found for the MnAl(im)/CTA-Mt samples is around 1 (Table 2), which confirms the lower average oxidation state of Mn in these materials and points to the beneficial role of using Ti-IMEC_NH3_ for enhancing the content of easily reducible Mn species formed upon calcination of the MnAl(im) component. The composites obtained from Ti-IMEC_NaOH_ possess very different TPR characteristics. In the MnAl(im)/Ti-IMEC_NaOH_-II-450 sample a single, broad maximum centered around 400 °C is observed, clearly being an envelope of several overlapping effects, which indicates a heterogeneity of Mn species valencies and environments, of average oxidation state ca. +3, as shown by the H/Mn ratio of 1.1. After calcination at 600 °C a substantial change in redox properties is observed, clearly associated with the evolution of the phase composition and destruction of the clay layers. The characteristic feature of the MnAl(im)/Ti-IMEC_NaOH_-II-600 material is a remarkable shift of TPR maxima towards higher temperature. The H/Mn ratio of 1 corresponds formally to Mn_2_O_3_-like stoichiometry. Comparison with TPR curves of other composites shows a virtual lack of easily reducible component, attributed in other materials to the reduction of Mn^4+^ species. In view of the XRD detected presence of Mn_3_O_4_ phase in this sample, the maximum around 500 °C may be attributed to the reduction of this phase to MnO, while the broad effect preceding this maximum shows that some more reducible MnO_x_ species, probably in less ordered environment, are present as well. Bearing in mind the XRD evidence of clay lattice destruction, accompanied by textural collapse, the strong second maximum, located at a much higher temperature than any of the effects found in other composites, suggests that it is due to manganese contained in another, amorphous phase, formed during solid component softening with the flux effect of sodium. To comply with the overall hydrogen consumption, manganese is expected to be present in this phase as Mn^3+^. It should be mentioned that the detrimental effect of sodium addition on the reducibility and textural properties of the MnO_x_/TiO_2_ system has been reported previously [40].

**Figure 6:**
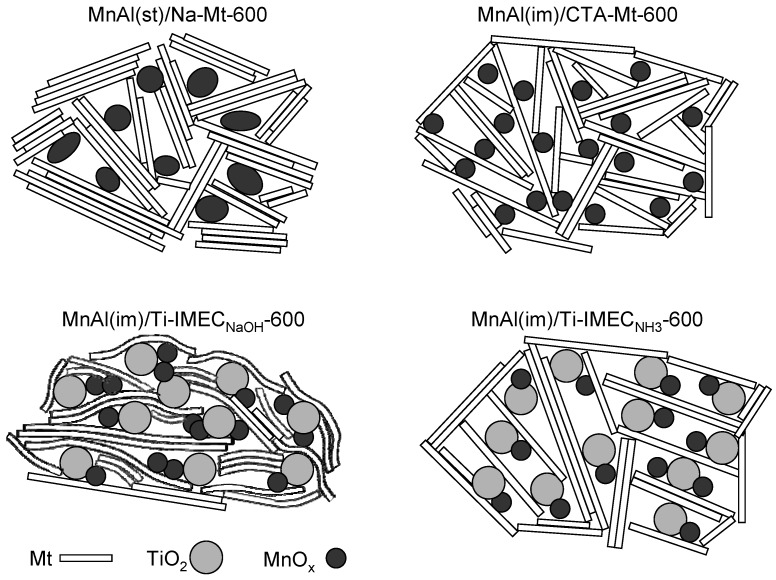
Schematic models of investigated composites calcined at 600 °C.

Thus, on the basis of the physico-chemical characterization one may conclude that in the case of TiO_2_-free composites, the use of an organic route yields MnAl(im)/CTA-Mt material of much better developed textural properties and excellent thermal stability, but characterized by a lower average oxidation state of Mn than in the MnAl(st)/Na-Mt sample obtained by the inorganic preparative procedure. The effect of titania addition depends whether NaOH or NH_3_ solutions are used in the Ti-IMEC neutralization step. The catalysts based on Ti-IMEC_NH3_ are characterized by the presence of a pretty uniform and thermally stable mesoporous network, and high abundance of easily reducible Mn species. In contrast, the composites derived from Ti-IMEC_NaOH_ are the least reducible of all studied composites and possess more heterogeneous porosity, which due to the presence of sodium, suffers thermal collapse upon increase of the calcination temperature from 450 to 600 °C. The schematic models of the composites structure after treatment at 600 °C are shown in Figure 6.

### 3.2. Catalytic Testing

**Figure 7:**
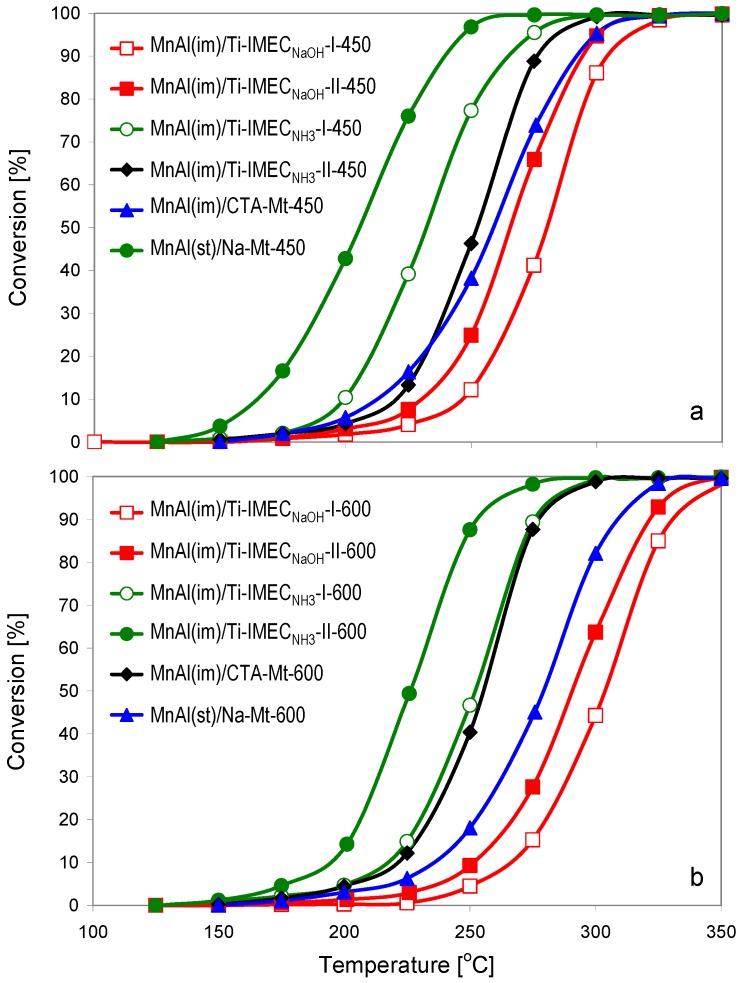
Ignition curves for toluene combustion over investigated composites calcined at (**a**) 450 °C; (**b**) 600 °C.

Catalytic activity of the composites was tested in the reaction of total combustion of toluene, frequently used as a model volatile organic compound. Figure 7a,b shows the toluene ignition curves of the composites calcined at 450 and 600 °C, respectively. Table 2 provides information on the temperatures at which 50% (T_50_) and 90% (T_90_) toluene conversion is reached for each of the catalysts. 

Analysis of Figure 7 shows that all investigated catalysts are characterized by very high activity, but differences are observed depending on the nature of the composite. Thus, it is evident that the MnAl(im)/CTA-Mt catalyst prepared by the organic route, performs better than the MnAl(st)/Na-Mt one, obtained by the inorganic procedure with conventionally synthesized Ht. This is especially visible when comparing performance after calcination at 600 °C, which, as revealed by the physico-chemical characterization has a negligible effect on the MnAl(im)/CTA-Mt material, while the MnAl(st)/Na-Mt is much more strongly affected, both in terms of structure, texture, and reducibility. In consequence, the MnAl(im)/CTA-Mt composite is a catalyst whose activity is practically independent on the calcination temperature, and the values of Δ_50_ = T_50_^600^ − T_50_^450^ and Δ_90_ = T_90_^600^ − T_90_^450^ are only 3 °C and 2 °C, respectively (Table 2). The relevant values for the MnAl(st)/Na-Mt catalyst equal 20 and 17 °C, which clearly shows the advantage of trapping MnAl(im) Ht nanoparticles between the organoclay layers, over the more conventional synthesis route. The enhanced stability of MnO_x_ phase generated from precursor embedded in organoclay is attributed to the fact that during calcination the combustion of interlayer CTA cations causes an additional increase of local temperature. The maximum of the exothermic effect associated with the combustion of organic matter appears around 300 °C [19], i.e., lower than any of the employed calcination temperatures. In consequence, the MnO_x_ component evolving during calcination experiences a temperature higher than the nominal one, which hardens the formed oxide against thermal degradation. 

For both temperatures of calcination the highest activity is observed for the Ti-IMEC_NH3_ based composites, which show spectacular combustion performance, the ones with higher MnAl(im) loading, MnAl(im)/Ti-IMEC_NH3_-II-450 and MnAl(im)/Ti-IMEC_NH3_-II-600, reaching 90% toluene conversion at 239 and 252 °C, respectively. This can be explained in terms of the catalysts’ physico-chemical characteristics, which show that the materials combine two features important for the catalytic reaction: good and relatively stable textural properties and a high contribution of easily reducible manganese component. It should be noted that according to the TPR data, incorporation of titania into the composite improves the reducibility of the MnO_x_ phase formed upon calcination of the MnAl(im) component. This is in accordance with previous reports indicating that titania-supported MnO_x_ catalysts exhibited lower reduction temperatures than the unsupported forms [41,42]. However, the performance of Ti-IMEC_NaOH_-based catalysts is inferior not only to the Ti-IMEC_NH3_-derived ones but also to the titania-free catalysts. Comparison of the data in Table 2 shows that not only are the absolute values of T_50_ and T_90_ significantly higher for catalysts obtained from Ti-IMEC_NaOH_ than for those prepared with Ti-IMEC_NH3_, but the former are characterized by higher Δ_50_ = T_50_^600^ − T_50_^450^ and Δ_90_ = T_90_^600^ − T_90_^450^ differences, which shows that they are more strongly affected by the increase of the temperature of calcination. This concerns in particular the Δ_90_ = T_90_^600^ − T_90_^450^ values, which are much lower for MnAl(im)/Ti-IMEC_NH3_-I and MnAl(im)/Ti-IMEC_NH3_-II, due to the steeper profiles of their light-off curves observed at 600 °C. The difference between MnAl(im)/Ti-IMEC_NaOH_-II-450 and MnAl(st)/Na-Mt-450, containing a comparable amount of Mn, is not very large. Since both catalysts display similar specific surface areas, the lower activity of the former appears to be related chiefly to the lower average oxidation state of the Mn species and their higher reduction temperature. After calcination at 600 °C, the difference becomes more pronounced, which reflects the effect of the collapsed porous network in MnAl(im)/Ti-IMEC_NaOH_-II-600, and further deterioration of the active phase reducibility. Thus, the presence of sodium in the composites obtained from Ti-IMEC_NaOH_, shown to affect their structural, textural and redox properties, has also a profound adverse effect on their catalytic properties. In contrast, the use of Ti-IMEC_NH3_ in combination with MnAl(im) component leads to composite catalysts of excellent combustion activity. Comparison with the literature data shows that in toluene combustion these materials perform considerably better than other previously described clay-transition metal oxide composite catalysts, e.g. Cu,Ce/Zr-PILC (T_90_ = 300 °C) [10], Fe-PILC (T_90_ = 380 °C) [15], or Fe/Ti-PILC (T_90_ = 347 °C) [18].

## 4. Conclusions

Physico-chemical characterization of novel clay/TiO_2_/MnAl-mixed oxide composites, in which non-clay components are obtained by an inverse microemulsion method, reveals profound differences in structural, textural, and redox properties of the materials, depending on the presence of the TiO_2_ component, the manner of titania nanoparticles preparation, and the temperature of calcination. Thus, the trapping of MnAl Ht nanoparticles, obtained from inverse microemulsion, between the organoclay layers, yields, after calcination, composite material of much better textural properties and with excellent thermal stability, but a lesser average oxidation state of Mn than the analogical material prepared from conventionally synthesized MnAl Ht and the sodium form of montmorillonite. The effect of insertion of MnAl Ht nanoparticles into clay, previously loaded with microemulsion containing TiO_2_ precursor, depends whether NaOH or NH_3_ solutions are used at the neutralization step. The materials obtained with the use of ammonia are characterized by the presence of a pretty uniform and thermally stable mesoporous network, coupled with a high abundance of easily reducible Mn species. In particular, their reducibility is better than that of composites without titania. In contrast, the composites derived from NaOH treated TiO_2_ precursor are significantly less reducible and possess more heterogeneous porosity, which, due to the presence of sodium, suffers thermal collapse upon increase of the calcination temperature from 450 to 600 °C. Catalytic tests of toluene combustion reveal that the clay/TiO_2_/MnAl-mixed oxide composites prepared with the use of ammonia show excellent activity, the composites obtained from MnAl Ht nanoparticles trapped between the organoclay layers are less active, but display spectacular thermal stability, while the clay/TiO_2_/MnAl-mixed oxide materials obtained with aid of NaOH are least active. The observed patterns of catalytic activity bear a direct relation to the structural, textural, and redox properties of the materials. 

## Figures and Tables

**Table 1:** XRF (X-ray fluorescence) determined chemical composition of the composites.

Sample	SiO_2_ [wt%]	Al_2_O_3_ [wt%]	MgO [wt%]	TiO_2_ [wt%]	MnO [wt%]	Fe_2_O_3_ [wt%]	Na_2_O [wt%]
MnAl(im)/Ti-IMEC_NaOH_-I	30.3	12.3	1.6	38.3	7.3	1.3	7.8
MnAl(im)/Ti-IMEC_NaOH_-II	24.9	12.4	1.4	33.0	18.2	1.2	7.9
MnAl(im)/Ti-IMEC_NH3_-I	33.7	13.2	1.8	41.5	7.2	1.5	-
MnAl(im)/Ti-IMEC_NH3_-II	28.5	13.8	1.5	36.6	17.5	1.3	-
MnAl(im)/CTA-Mt	49.2	26.0	2.9	-	18.5	2.3	-
MnAl(st)/Na-Mt	47.7	25.1	2.7	-	19.8	2.3	2.0

**Table 2:** S^BET^—specific surface area (in brackets % of the 450 °C specific surface retained at 600 °C), V_tot_—total pore volume, D^av^—average pore diameter, type of hysteresis loop, H/Mn—hydrogen consumption from TPR experiments, T_50_—temperature of 50% conversion, T_90_—temperature of 90% conversion, ΔT_50_—difference between T_50_ after calcination at 600 and 450 °C, and ΔT_90_—difference between T_90_ after calcination at 600 and 450 °C.

Sample	S_BET_ [m^2^/g]	V_tot_ [cm^3^/g]	D^av^ [nm]	Loop	H/Mn	T_50_ [°C]	T_90_ [°C]	ΔT_50_ [°C]	ΔT_90_ [°C]
MnAl(im)/Ti-IMEC_NaOH_-I-450	81	0.25	12.25	H3	-	280	304	24	27
MnAl(im)/Ti-IMEC_NaOH_-I-600	39 (48%)	0.20	20.72	H3	-	304	331		
MnAl(im)/Ti-IMEC_NaOH_-II-450	90	0.28	12.53	H3	1.1	265	295	26	27
MnAl(im)/Ti-IMEC_NaOH_-II-600	36 (40%)	0.24	25.94	H3	1.0	291	322		
MnAl(im)/Ti-IMEC_NH3_-I-450	161	0.23	5.72	H3/H5	-	232	265	20	10
MnAl(im)/Ti-IMEC_NH3_-I-600	108 (67%)	0.22	8.09	H3/H5	-	252	275		
MnAl(im)/Ti-IMEC_NH3_-II-450	178	0.28	6.28	H5	1.6	206	239	21	8
MnAl(im)/Ti-IMEC_NH3_-II-600	123 (69%)	0.26	8.58	H5	1.4	225	252		
MnAl(im)/CTA-Mt-450	132	0.33	10.08	H3	1.1	252	275	3	2
MnAl(im)/CTA-Mt-600	125 (95%)	0.36	11.39	H3	1.0	255	277		
MnAl(st)/Na-Mt-450	87	0.18	8.28	H3	1.5	259	292	20	17
MnAl(st)/Na-Mt-600	59 (68%)	0.19	12.88	H3	1.3	279	309		
